# Adiponectin, retinol-binding protein 4, and leptin in protracted critical illness of pulmonary origin

**DOI:** 10.1186/cc7956

**Published:** 2009-07-09

**Authors:** Lies Langouche, Sarah Vander Perre, Jan Frystyk, Allan Flyvbjerg, Troels Krarup Hansen, Greet Van den Berghe

**Affiliations:** 1 Department of Intensive Care Medicine, Katholieke Universiteit Leuven, Herestraat 49, 3000 Leuven, Belgium; 2 The Medical Research Laboratories, Clinical Institute, Aarhus University Hospital, Nørrebrogade 42-44, 8000 Aarhus, Denmark; 3 Immunoendocrine Research Unit, Medicial Department M, Aarhus University Hospital, Norrebrogade 42-44, 8000 Aarhus, Denmark

## Abstract

**Introduction:**

Critically ill patients requiring intensive care uniformly develop insulin resistance. This is most pronounced in patients with sepsis. Recently, several hormones secreted by adipose tissue have been identified to be involved in overall insulin sensitivity in metabolic syndrome-related conditions. However, little is known about these adipokines in critical illness.

**Methods:**

We studied circulating levels of the adipokines adiponectin, retinol-binding protein 4 (RBP4), and leptin during critical illness, and the impact of intensive insulin therapy, a therapy shown to affect insulin sensitivity, in serum samples from prolonged critically ill patients with a respiratory critical illness (n = 318). For comparison, we studied healthy subjects (n = 22) and acutely stressed patients (n = 22).

**Results:**

During acute critical illness, circulating levels of adiponectin, RBP4, and leptin were low. Patients with sepsis had lower levels of leptin and RBP4 than did nonseptic patients. When critical illness was sustained, adipokine levels returned to normal reference values. Insulin therapy enhanced adiponectin, blunted the rise of RBP4, and did not alter leptin levels.

**Conclusions:**

Acute critical illness is associated with immediate, but transiently low serum adipokine levels. Adiponectin and RBP4 are associated with altered insulin resistance in critical illness.

## Introduction

Critically ill patients requiring intensive care uniformly develop hyperglycemia in the presence of hyperinsulinemia, suggesting insulin resistance. This is most pronounced in patients with sepsis [1].

Recently, several hormones secreted by adipose tissue have been identified to be involved in overall insulin sensitivity in metabolic syndrome-related conditions. Adiponectin appears to increase insulin sensitivity, with low levels observed in conditions of insulin resistance, such as obesity and type 2 diabetes, and with higher levels being associated with increased insulin sensitivity [2,3]. Leptin and retinol-binding protein-4 (RBP4), the only specific transporter for retinol in the circulation, appear to affect insulin action. Circulating levels of RBP4 are elevated in subjects with obesity and type 2 diabetes and lower with improved insulin sensitivity [4,5]. Low leptin levels are present with insulin resistance, and insulin infusion can induce leptin secretion [6].

In insulin resistance of the critically ill, little is known about these adipokines. Furthermore, the impact of insulin therapy during critical illness on these adipokines has not been studied. We hypothesized that the insulin resistance that is present in critical illness would affect circulating adiponectin, RBP4, and leptin levels and that the improved insulin sensitivity that we observed in intensive insulin therapy (IIT) patients [7] would change the adipokine levels.

## Materials and methods

### Study design

This study was a subanalysis of a large (n = 1,200) prospective, randomized controlled study on the effects of IIT on outcome of critical illness [8]. The detailed protocol of the study was previously published [8]. Written informed consent was obtained from the closest family member. The protocol and consent forms, including later analyses, were approved by the Institutional Ethical Board. Patients randomly assigned to conventional insulin therapy (CIT) received insulin only when glucose concentrations exceeded 215 mg/dl, resulting in mean blood glucose of 153 mg/dl (hyperglycemia). IIT maintained blood glucose levels between 80 and 110 mg/dl, resulting in mean blood glucose of 111 mg/dl (normoglycemia). Caloric intake was not different between the two therapy groups. To reduce the number of samples for the current study, we chose to work with the largest homogeneous subset (43%) of the 1,200 originally included patients, identifiable on admission, being the subset of 512 patients with a respiratory disease as main reason for admission to the ICU. From those, we studied the 318 patients requiring at least 5 days of intensive care to assess the impact of IIT within the time frame that was required to bring about clinical benefits (Table 1). In this selection of long-stay respiratory patients, 100% of the patients assigned to IIT received insulin during their ICU stay (mean daily insulin dose, 83.1 ± 4.9 IU (mean ± SEM)), whereas 86.2% of the patients assigned to CIT received insulin (mean daily insulin dose, 25.3 ± 3.0 IU). In this selection of long-stay respiratory patients, all baseline characteristics except age were comparable in the two treatment populations (Table 1). IIT significantly reduced maximal Sequential Organ Failure Assessment (SOFA) score (indicating reduced organ failure), length of ICU stay, and in-hospital mortality (Table 1); no difference was seen in the cause of death in the ICU. For comparison, we studied, after informed consent, 22 overnight-fasted healthy volunteers (mean ± SD; age, 69 ± 8 years; BMI, 26.8 ± 3.5; 15 males), and 22 matched, not critically ill patients who underwent elective abdominal surgery (mean ± SD; age, 69 ± 13 years; BMI, 25.1 ± 2.6; 14 males). Blood samples taken from patients under acute surgical stress were obtained at the end of the procedure, before skin closure. After sampling and centrifugation, serum was kept frozen at -80°C until analysis. The protocols for both control studies were approved by the Institutional Ethical Board.

**Table 1:** Baseline and outcome characteristics of critically ill patients (ICU stay ≥5 days)

	CIT (n = 152)	IIT (n = 166)	*P*
Sex (number [percentage] male)	98 [64.5]	107 [64.5]	>0.9
Age (years; mean ± SD)	69 ± 14	64 ± 15	0.008
BMI (mean ± SD)	24.2 ± 5.2	24.7 ± 5.1	0.5
Adm. Apache II score (median [IQR])	23 [17.5–28.5]	22 [17–28]	0.4
Adm. SOFA score (median [IQR])	7 [4–9]	6 [4–8]	0.3
Kidney failure on admission (number [percentage])	23 [15.1]	23 [13.9]	0.7
History of diabetes (number [percentage])	16 [10.5]	23 [13.9]	0.4
Blood glucose on admission (mg/dl; mean ± SD)	167 ± 68	165 ± 68	0.7

Maximum SOFA score during ICU stay (mean ± SD)	10.5 ± 4.5	9.7 ± 4.3	0.05
Days in ICU (median [IQR])	14 [8–22]	10 [16–17]	0.008
Death in ICU (number [percentage])	60 [39.5]	50 [30.1]	0.08
In-hospital deaths (number [percentage])	90 [59.2]	71 [42.8]	0.003
Newly acquired kidney injury (number [percentage])	23 [15.1]	16 [9.6]	0.1

### Circulating adiponectin, RBP4, and leptin

Serum total adiponectin was measured with time-resolved immunofluorometric assay based on reagents from R&D Systems (Minneapolis, MN, USA). Serum RBP4 and serum leptin were determined with ELISA (Phoenix Pharmaceuticals, Burlingame, CA, USA) and RIA (Linco Research, Billerica, MA, USA), respectively. For all assays the intra- and interassay coefficients of variations (CVs) were less than 5% and 10%, respectively.

### Statistical analysis

The results were compared with unpaired Student's *t*-tests and Mann-Whitney *U *tests. We used the χ^2^-test for comparison of proportions. The significance of correlations between parameters was assessed by the Pearson (R) correlation coefficient. Statistical significance was considered when two-sided *P *values were below or equal to 0.05. Stat View 5.0.1 was used.

## Results

**Figure 1:**
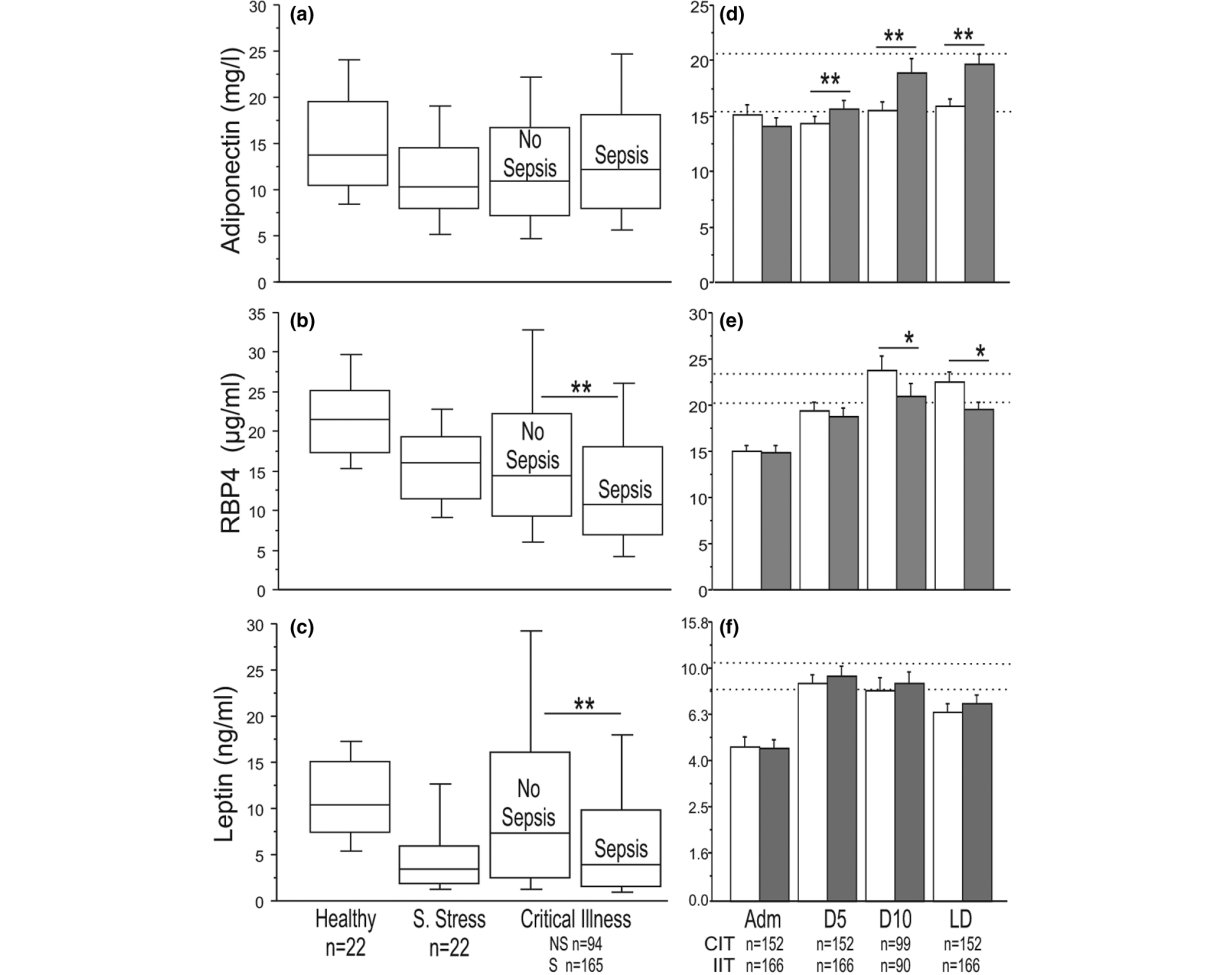
Circulating adipokines during critical illness. **(a-c) **Impact of acute illness: Results from healthy volunteers, patients not critically ill undergoing elective surgery, and critically ill patients on admission to the ICU are presented as box plots (boxes are medians and interquartile ranges; whiskers are 10^th ^and 90^th ^percentiles). **(d-f) **Critically ill patients who received CIT (white bars) or IIT (gray bars). Reference values (mean ± SEM) of healthy controls are indicated by two horizontal dotted lines. Adm = admission day; D5 = day 5; D10 = day 10; LD = the last day of ICU stay; ICU = intensive care unit. Data are presented as mean ± SEM. *P ≤ 0.05; **P ≤ 0.01. For statistical analysis, we subtracted corresponding admission-day values, and for leptin, we used log-transformed data as indicated on the figure.

Circulating adiponectin, RBP4, and leptin levels in the critically ill patients were low on admission to ICU as compared with those in healthy subjects (Figure 1). For RBP4, 38%, and for leptin, 27% of the admission values were below the lower limit of the 95% confidence interval of the levels observed in healthy volunteers. Patients undergoing elective surgery demonstrated lower serum adiponectin, RBP4, and leptin than did healthy controls (Figure 1). For RBP4, 20%, and for leptin, 24% of the values were below the lower limit of the 95% confidence interval of the levels observed in healthy volunteers. Critically ill patients with sepsis on ICU admission had even lower circulating admission levels of RBP4 and leptin than did patients without sepsis (Figure 1). Baseline patient characteristics of septic versus nonseptic patients were not different, except for the admission SOFA score (Table 2).

**Table 2:** Baseline and outcome characteristics of septic versus nonseptic critically ill patients (ICU stay ≥5 days)

	Proven sepsis (n = 165)	Proven no sepsis (n = 94)	*P*
Sex (number [percentage] male])	111 [67.3]	59 [62.8]	0.5
Age (years; mean ± SD)	66.1 ± 12.3	67.0 ± 15.2	0.6
BMI (mean ± SD)	25.1 ± 5.1	24.1 ± 5.0	0.1
Admission Apache II score (median [IQR])	23 [17–29]	21 [17–28]	0.6
Admission SOFA score (median [IQR])	7 [4–9]	4 [3–7]	< 0.0001
Kidney failure on admission (number [percentage])	27 [16.3]	12 [12.8]	0.4
History of diabetes (number [percentage])	20 [12.1]	15 [16.0]	0.4
Blood glucose on admission (mg/dl; mean ± SD)	164.5 ± 66.6	166.7 ± 70.3	0.8

Maximum SOFA score during ICU stay (mean ± SD)	9.4 ± 4.0	7.1 ± 3.7	< 0.0001
Days in ICU (median [IQR])	12 [7–20]	12.5 [7–19]	0.5
Death in ICU (number [percentage])	54 [32.7]	24 [25.5]	0.2
In-hospital deaths (number [percentage])	85 [51.5]	41 [43.6]	0.2

All subjects were screened for infection on admission and were defined retrospectively for this *post hoc *study as "*proven *sepsis" when infection was *proven *and the Bone criteria were fulfilled [29]. Patients *proven *not to have sepsis with this strict definition were classified as "*proven *no sepsis". Fifty-nine patients for whom treatment with antibiotics was started before admission to the ICU were categorized as unknown and therefore excluded from this subanalysis.

With time in ICU, circulating levels of all three adipokines increased, although leptin remained low in septic patients throughout their stay in the ICU (*P *= 0.007 for day 5; *P *= 0.06 for day 10; and *P *= 0.002 for the last day). Neither adiponectin, leptin, nor RBP4 correlated significantly with the cytokines IL-6, IL-8, IL-10, and TNF-α.

On all studied time points, adiponectin and leptin levels were significantly higher in female subjects than in male subjects (data not shown) and correlated with BMI (*R *= -0.194; *P *= 0.0002 for adiponectin; *R *= 0.456; *P *< 0.0001 for leptin). No correlation was present between any of the studied adipokines and age.

IIT further increased the serum levels of adiponectin, whereas it blunted the rise of serum RBP4, with no effect on serum leptin (Figure 1). Circulating RBP4 correlated positively with serum creatinine levels throughout ICU stay (admission *R *= 0.363; day 5 *R *= 0.406; day 10 *R *= 0.584; last-day *R *= 0.475; *P *< 0.0001 for all). Serum leptin levels correlated positively with the corresponding circulating insulin levels (admission *R *= 0.373; day 10 *R *= 0.330; last-day *R *= 0.292; *P *< 0.0001 for all).

## Discussion

We observed low circulating levels of adiponectin, RBP4, and leptin in critically ill patients on admission to the ICU, with lowest values in patients with sepsis, but also in patients not critically ill under acute surgical stress. These observations would suggest an acute stress response. Low levels of circulating RBP4 and leptin were previously observed after burn injury or trauma or both [9,10], and low levels of adiponectin were reported in rats with sepsis [11]. It is unclear what mediates this acute lowering with stress. Theoretically, reduced synthesis or increased removal, or both, either by extravasation or by increased metabolic clearance, may play a role. The lower values in patients with sepsis would suggest the former, as this condition is characterized by capillary leakage, which could have removed the adipokines to the interstitial compartment. The low adipokine levels might in part be a consequence of the inactivity and malnourished status of the medical intensive care patients on admission to the ICU and of the overnight fasting of the patients undergoing elective surgery. Fasting reduces leptin levels [12], and severe calorie restriction with weight loss reduces circulating RBP4 [13]. However, an increase in circulating adiponectin levels would be expected [14], and thus, this cannot explain the changes in the ICU patients, particularly because the healthy volunteers were fasted overnight.

We found adiponectin and leptin levels to be higher in women than in men and correlating with the BMI. This gender difference has been related to the difference in sex steroids and to the higher ratio of subcutaneous to omental fat mass in women [15].

IIT targeted to normoglycemia further increased the rise of serum adiponectin with time in the ICU. This corresponds with the altered insulin sensitivity that has been associated with better glycemic control in patients with type 2 diabetes [16,17], but also in critically ill patients treated with IIT [2,7]. IIT blunted the rise in serum RBP4 that occurs with time in ICU. Decreasing levels of RBP4 were associated previously with altered insulin resistance [4,5]. Euglycemia with insulin therapy in severely burned children also decreased circulating RBP4 [18]. However, some contrasting results were reported regarding the association between circulating RBP4 and insulin resistance [19,20]. Furthermore, increased levels of circulating RBP4 have been described in patients with chronic renal failure and attributed to reduced glomerular filtration [21].

In our setting, IIT improved kidney function [8,22], and circulating RBP4 levels correlated with creatinine levels. Leptin levels are known to correlate with reduced insulin sensitivity, and insulin infusion can induce leptin secretion [6,23]. However, IIT failed to change leptin levels throughout the ICU stay. This can be explained by the observation that, although several-fold higher insulin doses were required to maintain normoglycemia with IIT, serum insulin levels were equal between the two therapy groups [24] and correlated well with leptin at all studied time points. Furthermore, it was reported that C-reactive protein, which reaches very high levels in critical illness, inhibits leptin binding to its receptor and blocks cellular signaling [25].

We must highlight two shortcomings in our study. As we studied only those patients with a respiratory disease as the main reason for admission to the ICU, extrapolation of our results to other diagnostic categories should be done with great caution. We studied adipokine levels only in prolonged critically ill patients, as our primary objective was to assess the role of the studied adipokines on the impact of IIT within the time frame that was required to bring about clinical benefits. Hence, an early effect may have been missed in this study. Earlier studies on leptin levels in the acute phase (first 24 hours) of sepsis describe a sepsis-induced elevation in leptin [26-28]. The elevation does not appear to be driven by the severity of illness, as survivors showed higher levels than did nonsurvivors [26-28]. These results and the observations that diet, insulin, BMI, gender, and cytokines may affect leptin levels make it clear that the role of leptin in critical illness remains incompletely understood.

## Conclusions

Acute stress, caused by surgery or by critical illness of pulmonary origin, was associated with immediate but transiently low serum adiponectin, leptin, and RBP4 levels. Sepsis especially reduced RBP4 and leptin levels. IIT accentuated the rise of circulating adiponectin levels occurring with time in the ICU, blunted the rise of RBP4, and did not alter leptin levels. The effects of IIT on adiponectin and RBP4 are indicative of altered insulin sensitivy with IIT in the ICU patients.

## Key messages

• Acute surgical or medical stress were associated with transiently lowered levels of the circulating adipokines adiponectin, RBP4, and leptin.

• Sepsis was associated with low circulating leptin levels throughout an intensive care stay.

• Intensive insulin therapy increased circulating adiponectin and blunted circulating RBP4, indicative of altered insulin sensitivity.

## Abbreviations

CIT: conventional insulin therapy; IIT: intensive insulin therapy; RBP4: retinol-binding protein 4.

## Competing interests

The authors declare that they have no competing interests.

## Authors' contributions

LL, SVDP, JF, and AF contributed to acquisition of the data; LL, TKH, and GVDB participated in the design, coordination, and statistical analysis. LL and GVDB drafted the manuscript. All authors read and approved the final manuscript.
